# CX08005, a Protein Tyrosine Phosphatase 1B Inhibitor, Attenuated Hepatic Lipid Accumulation and Microcirculation Dysfunction Associated with Nonalcoholic Fatty Liver Disease

**DOI:** 10.3390/ph16010106

**Published:** 2023-01-11

**Authors:** Jiang Li, Xiaolin Zhang, Jinying Tian, Juan Li, Xuechen Li, Song Wu, Yuying Liu, Jingyan Han, Fei Ye

**Affiliations:** 1 Beijing Key Laboratory of New Drug Mechanisms and Pharmacological Evaluation Study, Institute of Materia Medica, Chinese Academy of Medical Science & Peking Union Medical College, 1 Xiannongtan St., Beijing 100050, China; 2 Diabetes Research Center of Chinese Academy of Medical Sciences and Peking Union Medical College, Beijing 100730, China; 3 Key Laboratory of Microcirculation, State Administration of Traditional Chinese Medicine of the People’s Republic of China, Beijing 100191, China; 4 Department of Integration of Chinese and Western Medicine, School of Basic Medical Sciences, Peking University, Beijing 100191, China

**Keywords:** CX08005, PTP1B inhibitor, insulin sensitivity, microcirculation, NAFLD

## Abstract

Nonalcoholic fatty liver disease (NAFLD) is one of the common metabolic diseases characterized by hepatic lipid accumulation. Insulin resistance and microcirculation dysfunction are strongly associated with NAFLD. CX08005, an inhibitor of PTP1B with the IC_50_ of 0.75 ± 0.07 μM, has been proven to directly enhance insulin sensitivity. The present study aimed to investigate the effects of CX08005 on hepatic lipid accumulation and microcirculation dysfunction in both KKAy mice and diet-induced obesity (DIO) mice. Hepatic lipid accumulation was evaluated by hepatic triglyceride determination and B-ultrasound analysis in KKAy mice. Insulin sensitivity and blood lipids were assessed by insulin tolerance test (ITT) and triglyceride (TG)/total cholesterol (TC) contents, respectively. In addition, the hepatic microcirculation was examined in DIO mice by in vivo microscopy. The results showed that CX08005 intervention significantly reduced the TG and echo-intensity attenuation coefficient in the livers of KKAy mice. Furthermore, we found that CX08005 treatment significantly enhanced insulin sensitivity, and decreased plasma TG and/or TC contents in KKAy and DIO mice, respectively. In addition, CX08005 treatment ameliorated hepatic microcirculation dysfunction in DIO mice, as evidenced by increased RBCs velocity and shear rate of the blood flow in central veins and in the interlobular veins, as well as enhanced rate of perfused hepatic sinusoids in central vein area. Additionally, CX08005 administration decreased the adhered leukocytes both in the center veins and in the hepatic sinusoids area. Taken together, CX08005 exhibited beneficial effects on hepatic lipid accumulation and microcirculation dysfunction associated with NAFLD, which was involved with modulating insulin sensitivity and leukocyte recruitment, as well as restoration of normal microcirculatory blood flow.

## 1. Introduction

Non-alcoholic fatty liver disease (NAFLD), one of the most common metabolic diseases characterized by hepatic lipid accumulation [1], is a highly prevalent disease with an estimated prevalence rate of more than 25% in adult population [1,2,3]. There is an increased risk of developing into cirrhosis and liver cancer in NAFLD [4], which is a serious threat to health and a serious burden on families and society [5]. Evidence is accumulating that insulin resistance is strongly associated with NAFLD [6]. Apart from the classical metabolic effects, insulin has important non-metabolic hemodynamic effects [7,8,9]. Insulin resistance increases hepatic de novo lipogenesis [10], enhances adipose tissue lipolysis with subsequent elevation in circulating free fatty acids (FFA) [6], impairs the transcapillary passage of insulin to target tissues, and decreases microvascular blood flow and expansion of the capillary network [11]. Hepatic microcirculation dysfunction has been proven to play a critical role in NAFLD progression [12]. The reduction in microcirculatory blood flow is associated with the lipid accumulation and contributes to increased reactive oxygen species (ROS) production and lipid peroxidation in the liver [13,14]. Therefore, improving insulin resistance and microcirculation dysfunction are important strategies to prevent/delay the onset and progression of NAFLD.

Protein tyrosine phosphatase 1B (PTP1B) is a negative regulator of insulin/leptin signal transduction pathways. PTP1B catalyzes the dephosphorylation of the insulin receptor (IR) and leptin receptor-associated Janus kinase 2 (JAK2), respectively, which reduces insulin sensitivity and shuts down cytokine-mediated signaling pathways [15,16]. PTP1B inhibition may represent a promising drug target for NAFLD since its deficiency in mice enhanced insulin sensitivity and conferred resistance to diet-induced hepatic steatosis [17,18].

CX08005 is a highly selective and strongly competitive inhibitor of PTP1B with an IC_50_ of 7.81 × 10^−7^ M. Docking simulation analysis indicate that CX08005 bonds to PTP1B at the catalytic residue Cys^215^, and subsequently inhibits its phosphatase activity. It has been confirmed to directly enhance insulin sensitivity in diet-induced obese (DIO) and KKAy mice, as well as 3T3-L1 adipocytes, C2C12 myotubes, and HepG2 hepatocytes [19]. Nonetheless, whether CX08005 can improve the NAFLD remains to be elucidated. Therefore, in this study, we evaluated the effects of CX08005 on NAFLD-associated hepatic lipid accumulation and microcirculation dysfunction.

## 2. Results

### 2.1. Attenuated the Hepatic Lipid Accumulation in Mice

**Figure 1:**
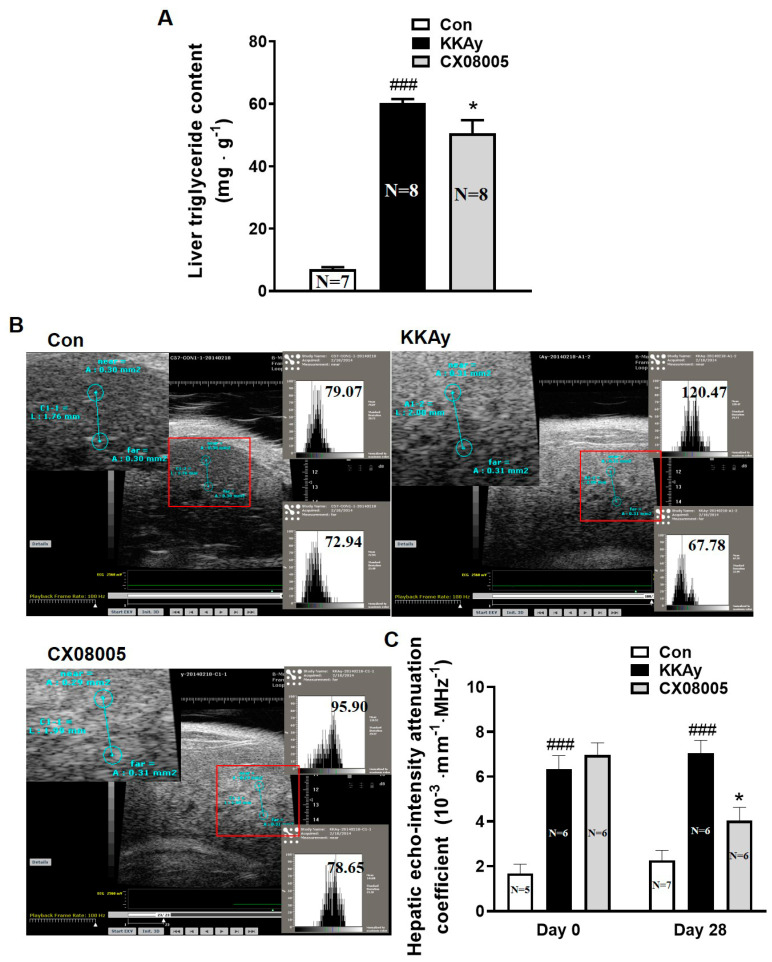
The effects of CX08005 on hepatic lipid accumulation in KKAy mice. (**A**) Quantitative analysis in hepatic triglyceride (N = 7–8). (**B**) Representative images of liver ultrasound after the treatment with CX08005 for 28 days. (**C**) Analysis of the hepatic echo-intensity attenuation coefficient after the treatment with CX08005 for 4 weeks (N = 5–7). Data are expressed as mean ± SEM ### *p <* 0.01 vs. Con; * *p <* 0.05 vs. KKAy.

Hepatic lipid accumulation is a hallmark of NAFLD. Distinct hepatic lipid accumulation was observed in KKAy mice. Compared with those in the age-matched C57BL mice (Con), the hepatic TG content was increased by 8.6-fold in KKAy mice (Figure 1A). After the oral treatment with CX08005 for 28 consecutive days, the hepatic lipid accumulation in KKAy mice was attenuated with a 16.5% drop of the hepatic TG content (Figure 1A). Meanwhile, liver B-ultrasound analysis was performed before and after CX08005 administration, respectively, to evaluate the hepatic lipid content (Figure 1B). As per the results, the hepatic echo-intensity attenuation coefficient in B-ultrasound analysis was enhanced by 2.8-fold (Figure 1B,C) in KKAy mice compared to the age-matched C57BL mice (Con). During the CX08005 treatment, the hepatic echo-intensity attenuation coefficient, self-comparison between day 0 and day 28, showed no obvious changes in the age-matched C57BL mice (Con) and in KKAy mice, but was decreased by 43% in KKAy mice with CX08005 treatment (Figure 1B,C).

### 2.2. Improved the Dyslipidemia in Mice

**Figure 2:**
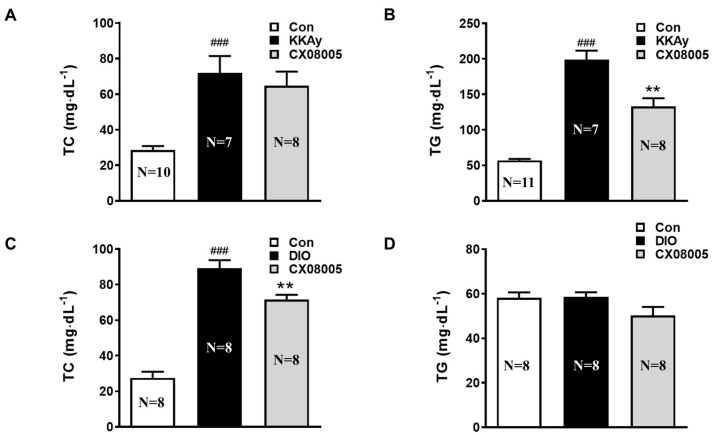
The effects of CX08005 on dyslipidemia in mice. After the treatment with CX08005 for 2 weeks, lipidemia determination were performed in both KKAy and DIO mice (N = 7–11). (**A**) Plasma cholesterol content in KKAy mice. (**B**) Plasma triglycerides content in KKAy mice. (**C**) Plasma cholesterol content in DIO mice. (**D**) Plasma triglycerides content in DIO mice. Data are expressed as mean ± SEM. ### *p <* 0.001 versus Con; ** *p <* 0.01 versus KKAy or DIO mice.

Usually, dyslipidemia, such as hypercholesterolemia and hypertriglyceridemia, is closely associated with NAFLD [10]. We then further investigated the effect of CX08005 on dyslipidemia in both KKAy and DIO mice, respectively. As the results, the plasma TG and TC levels in KKAy mice were significantly increased by 250% and 138%, respectively, compared to those in the age-matched C57BL mice (Con). After treatment with CX08005, the hyperlipidemia was improved with an obvious decrease of the TG level by 33%, and a downtrend of the TC level by 10% (Figure 2A,B). Moreover, in DIO mice, similar hypercholesterolemia was exhibited by 224% increase compared with that in the same batch of normal control mice (Con), and was reverted by CX08005 treatment by 20% in TC level (Figure 2C). For the plasma TG level, there was no significant alteration in DIO mice, and no obvious effect by CX08005 treatment was found (Figure 2D).

### 2.3. Effects on Insulin Response in Mice Targeting on PTP1B

**Figure 3:**
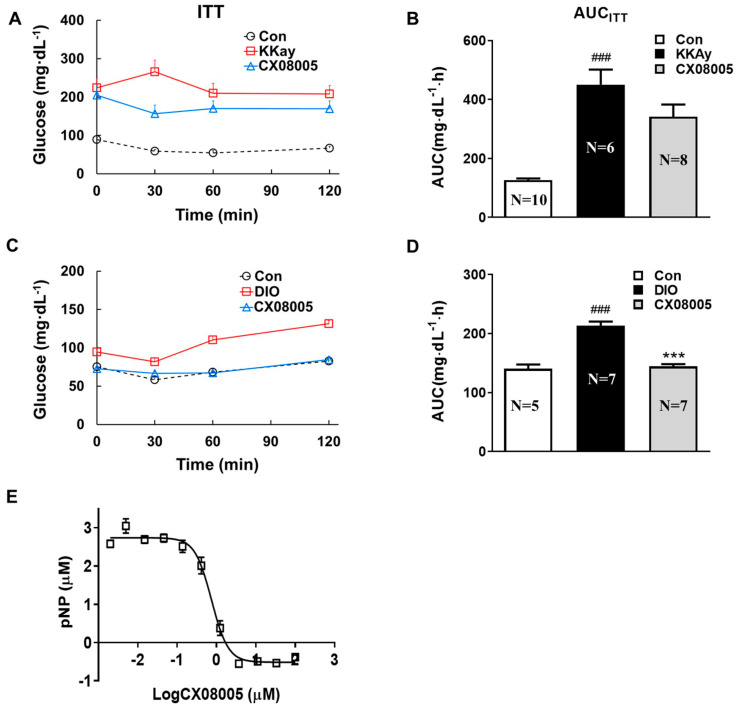
The effects of CX08005 on insulin resistance targeting on PTP1B. After the treatment with CX08005 for 2 weeks, insulin tolerance test was performed in both KKAy and DIO mice (N = 5−10). (**A**) Changes of blood glucose levels of ITT in KKAy mice. (**B**) Values of AUC_KKAy_. (**C**) Changes of blood glucose levels of ITT in DIO mice. (**D**) Values of AUC_DIO_. (**E**) IC_50_ value (N = 3). Data are expressed as mean ± SEM. ### *p <* 0.001 versus Con; *** *p <* 0.001 versus KKAy or DIO mice.

The compound CX08005 is identified as a PTP1B inhibitor and an insulin sensitizer in our previous study [19]. In the present study, KKAy mice exhibited significant insulin resistance, as evidenced by a 257% higher AUC_KKAy_ value of ITT relative to that of the age-matched C57BL mice (Con). After CX08005 (50 mg/kg) treatment for 2 weeks, the elevated AUC_KKAy_ was decreased by 24% in KKAy mice (Figure 3A,B). In addition, DIO mice had a blunted response to insulin, indicating insulin resistance as assessed by ITT, in which the parameter AUC_DIO_ in DIO mice was 52% higher than that of normal mice in the same batch (Con). After 2 weeks treatment by CX08005 (100 mg/kg), the levels of blood glucose were lowered, and the elevated AUC_DIO_ value was suppressed by 32% (Figure 3C,D), respectively, in ITT. Concurrently, the effects of CX08005 on PTP1B inhibition was also confirmed with the IC_50_ of 0.75 ± 0.07 μM (Figure 3E).

### 2.4. Ameliorating Hepatic Microcirculation Dysfunction in Mice

**Figure 4:**
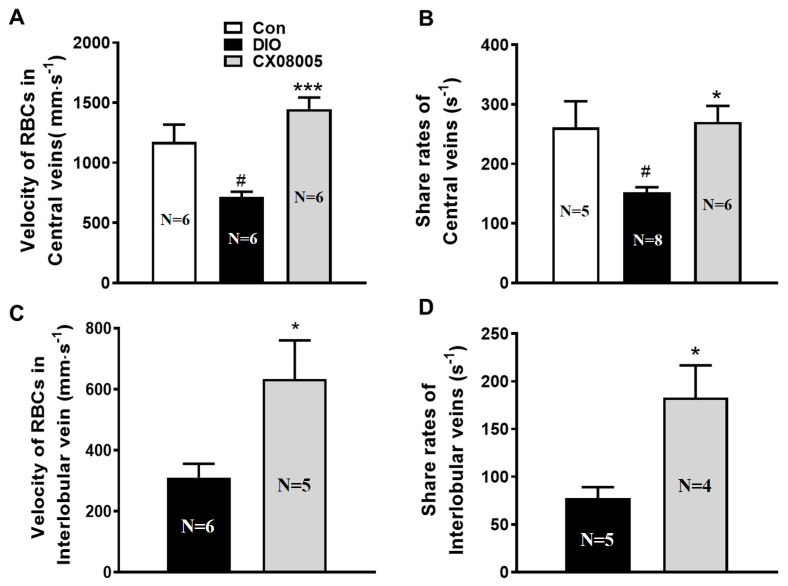
Effects of CX08005 on RBCs velocity and shear rates in DIO mice. (**A**) RBCs Velocity in central veins. (**B**) Shear rates of central veins. (**C**) RBCs Velocity in interlobular veins. (**D**) Shear rates of interlobular veins. Data are shown as means ± SEM. N = 4–8. # *p <* 0.05 vs. Con; * *p <* 0.05, *** *p <* 0.001 vs. DIO mice.

**Figure 5:**
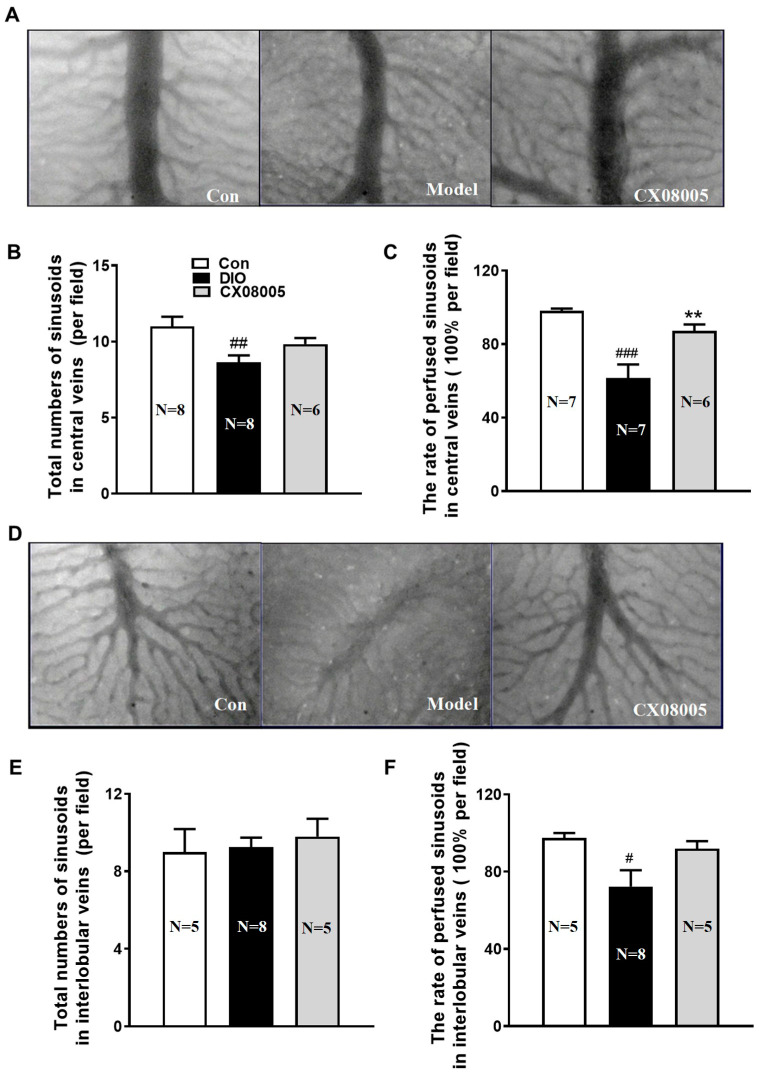
Effects of CX08005 on perfused sinusoids of central veins and interlobular veins in DIO mice. (**A**) Perfused sinusoids of central veins (×200). (**B**) Total numbers of sinusoids in central veins. (**C**) Rate of perfused sinusoids of central veins. (**D**) Perfused sinusoids of interlobular veins (×200). (**E**) Total numbers of sinusoids in interlobular veins. (**F**) Rate of perfused sinusoids of interlobular veins. Data are shown as means ± SEM. N = 5–8. # *p <* 0.05, ## *p <* 0.01, ### *p* < 0.001 vs. Con; ** *p <* 0.01 vs. DIO mice.

It has been shown that insulin resistance contributes to endothelial dysfunction [11]. Microcirculatory disturbance is often accompanied during the progress of NAFLD [20]. As the results, the microcirculation disorder also occurred in DIO mice which implicated obvious hepatic lipid accumulation. The hepatic microcirculation situation revealed decreased RBCs velocity (39%), shear rate (42%) (Figure 4A,B), the number of the total hepatic sinusoids (22%) (Figure 5B), as well as the rate of perfused hepatic sinusoids in the center (37%) (Figure 5C) and interlobular (28%) (Figure 5F) veins of the DIO mice compared to those of the same batch normal mice (Con). CX08005 treatment significantly increased the RBCs velocity and shear rate of the blood flow in central veins by 101% and 78%, respectively, compared to the DIO mice (Figure 4A,B). Consistent with the central veins, the RBCs velocity and shear rate in DIO mice were markedly ameliorated by 104% and 136% in the interlobular veins by CX08005 treatment (Figure 4C,D). Furthermore, CX08005 intervention increased the number of total hepatic sinusoids and the rate of perfused hepatic sinusoids in central vein area by 14% and 42%, respectively, relative to DIO mice (Figure 5A–C); whereas, these effects were not observed in the interlobular veins (Figure 5D–F). These results showed that CX08005 could ameliorate the hepatic microcirculatory disturbances in DIO mice.

### 2.5. Attenuating Adhesion of Leukocytes to the Venular Wall in Mice

Chronic inflammation is considered as a driver to the progression of NAFLD, and leukocyte recruitment to the venular wall plays a key role in the inflammatory response [21]. In this study, we further analyzed the effects of CX08005 on DIO-provoked leukocyte rolling and adhesion in mice hepatic venules.

**Figure 6:**
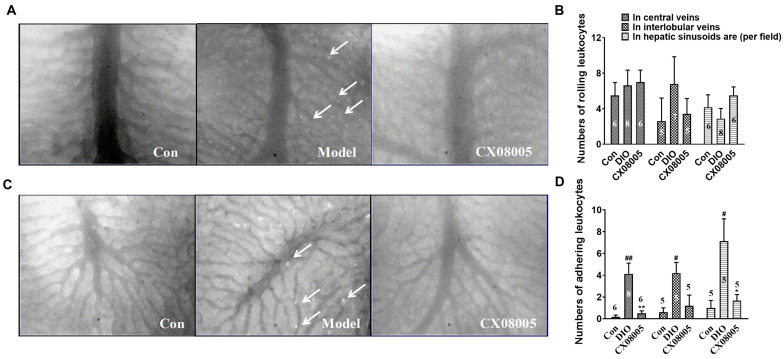
Effects of CX08005 on rolling and adhering leukocytes in DIO mice. (**A**) Adhering leukocytes in central veins (×200); (**B**) Numbers of rolling leukocytes; (**C**) Adhering leukocytes in interlobular veins (×200); (**D**) Numbers of adhering leukocytes. Data are shown as means ± SEM. N = 5–8. # *p <* 0.05, ## *p <* 0.01, vs. Con; * *p <* 0.05, ** *p <* 0.01 vs. DIO mice. White arrows indicate rolling/adherent leukocytes in central veins or interlobular veins.

Our results showed that there was no significant difference in the number of rolling leukocytes among three groups (Figure 6A,B). However, the number of adherent leukocytes was significantly increased in the center veins (25.5-fold), the interlobular veins (6.1-fold), and the hepatic sinusoids area (6.7-fold) in DIO mice (Figure 6C,D). CX08005 administration decreased the adhered leukocytes both in the center veins (89%) and in the hepatic sinusoids area (78%). These results suggested that CX08005 might be inhibited the inflammatory response by attenuating adhesion of leukocytes to the venular wall in DIO mice.

## 3. Discussion

NAFLD is strongly associated with insulin resistance, which disrupts glycolipid metabolism in hepatocytes, and is considered as a common pathophysiological basis of hepatic lipid accumulation and glycolipid metabolism [22,23]. On the other hand, dysregulated glycolipid metabolism promotes hepatic lipid accumulation, inflammatory reaction, and further leads to lipotoxic states, such as worse hepatic microcirculation, insulin resistance, etc. [23]. Therefore, NAFLD is often accompanied with dyslipidemia, insulin resistance, and microcirculation disorder in clinical practice [24,25].

As an ideal animal model, which is very close to the pathophysiological characters and its program of the clinical metabolic syndrome, KKAy mouse and DIO mouse has always been concerned [26,27,28]. KKAy mouse, characterized by insulin resistance, type 2 diabetes, hypercholesterolemia, hypertriglyceridemia, and NAFLD, is an animal model of polygenic inheritance with the congenital glycolipid disorders. A little different from the KKAy mouse, DIO mouse is induced by excessive energy intake and is usually characterized by insulin resistance, type 2 diabetes, hypercholesterolemia, and NAFLD, but less hypertriglyceridemia. Here, we used both KKAy mice and DIO mice, respectively, to study the effects of CX08005 on hepatic lipid accumulation and microcirculation dysfunction associated with NAFLD.

The liver biopsy is the gold standard to diagnose NAFLD. However, liver biopsy is invasive and may cause serious complications [29]. Early diagnosis of mild hepatic steatosis in NAFLD has important implications for evaluating the efficacy of NAFLD treatment. Liver ultrasound scan is one of the common methods for diagnosing NAFLD. The hepatic echo-intensity attenuation rate is an ultrasonic quantitative indicator, which is correlated with the degree of NAFLD in animal experiments [30]. In our laboratory, the hepatic echo-intensity attenuation coefficient has been validated and used to assess NAFLD in mice with a correlation coefficient of r = 0.744 [31]. It is an easy, impersonal, non-invasive method for the estimation of hepatic fat content. In this study, the hepatic TG and echo-intensity attenuation coefficient were used to estimate hepatic lipid accumulation in KKAy mice. We observed that KKAy mice presented distinct features of T2DM and/or NAFLD, including insulin resistance, developed disorders of glycolipid metabolism, hepatic lipid accumulation, as well as enhancement in hepatic echo-intensity attenuation coefficient, which was consistent with our previous results [32].

According to the classic “second strike” hypothesis of the NAFLD pathogenesis, “first strike” is liver steatosis, and “second strike” consists of the synergistic effect of multiple factors, such as insulin resistance, inflammatory, etc. Among them, insulin resistance is one of the key factors in the development of liver steatosis and NASH. Insulin resistance, leading to hyperinsulinemia, causes growing lipid accumulation in liver through the increase in de novo lipid synthesis in liver and the enhancement of fatty acid flow to liver induced by the impairment of lipolysis in adipose tissue [33,34]. Our previous studies showed that CX08005, a novel PTP1B inhibitor, had insulin-sensitizing effects in both KKAy and DIO mice [19]. The inhibition of PTP1B by CX08005 in vitro and the enhanced insulin responses in ITT of both KKAy and DIO mice were also confirmed in this study (Figure 3.). We further found that CX08005 improved hepatic lipid accumulation by decreasing 16.5% of the TG content in liver in KKAy mice. This phenomenon was also observed in B-ultrasound analysis by reducing hepatic echo-intensity attenuation coefficient to illustrate that CX08005 could ameliorate NAFLD in KKay mice (Figure 1B,C). We also observed that CX08005 treatment could significantly reverse hypertriglyceridemia in KKAy mice (Figure 2B.) and the hypercholesterolemia in DIO mice, respectively (Figure 2C). The results of this study suggest that CX08005 could improve lipid metabolic disorders associated with NAFLD through ameliorating insulin resistance, the major mechanism of CX08005. However, whether insulin resistance causes liver steatosis or NAFLD causes insufficient insulin degradation to promote hyperinsulinemia and secondary insulin resistance is still under discussion.

Insulin resistance also contributes to endothelial dysfunction and subsequent microcirculation disorders [11]. It was well known that insulin interacted with vascular endothelial insulin receptors and subsequently activated the PI3K/Akt signaling pathway, thereby activating the key enzyme endothelial nitric oxide (NO) synthase (eNOS)-mediated vasodilation and angiogenesis. Insulin resistance has been shown to contribute to endothelial dysfunction, manifested by impaired the transcapillary passage of insulin to target tissues, inadequate coronary dilation and/or abnormal vasoconstriction, and peripheral arterial microcirculation disturbances [7,8,9,11]. Furthermore, endothelial PTP1B itself also mediates endothelial dysfunction by impairing endothelial cell angiogenic responses through endoplasmic reticulum stress [35,36]. PTP1B depletion induces endothelium-dependent vasorelaxation in microvessels in diabetic animals [37,38]. On other hand, endothelial dysfunction can induce some panvascular diseases including insulin resistance and metabolic syndrome, such as NAFLD, diabetes, etc. [39,40,41]. It is well known that the reduction in microcirculatory blood flow is associated with the degree of steatosis and contributes to increased ROS production and lipid peroxidation in the liver, ultimately leading to tissue damage [13,14]. Our previous study also demonstrated substantial microvascular changes in DIO mice associated with NAFLD [32].

Therefore, in the present study, apart from affecting insulin resistance and lipid metabolic disorders, the protective effect of CX08005 against hepatic microcirculation dysfunction was observed by the inverted intravital microcirculation observation system in the liver of DIO mice. Diet-induced NAFLD with obesity and insulin resistance could rapidly develop liver inflammation, such as steatohepatitis and fibrosis. Leukocyte adhesion to the vasculature further exacerbates the activation of the inflammatory response [40]. Kupffer cells, tissue-fixed macrophages located in the sinusoids of the liver, represent the highest concentration of mononuclear phagocytes in the body. Activated Kupffer cells cause platelet and leukocyte adhesion to the sinusoidal endothelium promote inflammatory responses and oxidative stress, and stimulate hepatic stellate cells [42]. Furthermore, the activation of hepatic stellate cells is the main driving force for liver fibrosis [43]. Interestingly, in DIO mice, apart from increasing insulin sensitivity and improving dyslipidemia, CX08005 treatment reduced leukocyte recruitment and increased blood flow in the hepatic microcirculation. Therefore, we speculate that CX08005 treatment for NAFLD may be related to the inhibition of oxidative stress and the secretion of pro-inflammatory cytokines mediated by insulin sensitization and hepatic microcirculation recovery, and the specific mechanism needs to be further explored.

In addition, CX08005 was considered to be a safe agent with a tolerance dose of 1000 mg/kg once orally in the acute toxicity study of ICR mice, of 100 mg/kg in a single dosage orally in cardiovascular and respiratory safety pharmacology studies of beagle dogs (detailed data not shown).

Although PTP1B inhibition has been disclosed to have the potential for metabolic syndrome treatment. However, most PTP1B inhibitors lack specificity due to high conservation among protein tyrosine phosphatases (PTPs). To date, only a few PTP1B inhibitors have entered clinical studies. Unfortunately, most of them have been discontinued due to lack of specificity and serious side effects [44]. Our previous study showed that CX08005 has a similar IC_50_ value against PTP1B and T-cell protein tyrosine phosphatase (TCPTP), which is necessary to exclude a side effect, developing potent and selective inhibitors targeting on PTP1B would be a reasonable strategy in the future.

## 4. Materials and Methods

### 4.1. PTP1B Inhibitory Activity

In vitro enzymatic activity of PTP1B was measured by using 4-nitrophenyl phosphate disodium salt (pNPP) as substrate as described before [19]. Briefly, the compound and recombinant human His-PTP1B protein were pre-incubated at room temperature for 5 min. Assays were performed in 96-well plates with a final volume of 100 μL reaction buffer containing 50 mM HEPES, 5 mM DTT, 150 mM NaCl, 2 mM EDTA and 2 mM pNPP (pH 7.0), incubated at 30 °C for 10 min, stopped by addition of 50 μL 3 M NaOH. The absorbance density of p-nitrophenol was monitored at 405 nm. A similar system without His-PTP1B protein was used as blank. The effects of different dosages of CX08005 were measured, and the IC_50_ value was calculated with GraphPad Prism 5.0.

### 4.2. Animals and Drug Administration

Animals were obtained from HFK Bioscience Co., Ltd. (Beijing, China). KK-Ay mice (male, average weight ~35 g; 11 weeks old) were fed chow diet (1K65, Beijing HFK Bioscience Co., Ltd., Beijing, China). C57BL/6 mice (male, average weight ~14 g; 4 weeks old) were fed high-fat-diet (50% fat, 36% carbohydrate, and 14% protein in energy) for diet-induced obesity (DIO). Aged-matched male C57BL/6 mice fed with the standard chow diet (1022, Beijing HFK Bioscience Co., Ltd., Beijing, China, containing 12% fat, 62% carbohydrate, and 26% protein in energy) were employed as normal control (Con).

Study in KKAy mice. KKAy mice were randomly divided into two groups: the model control group (KKAy) and treated group. Treated group was received CX08005 (50 mg/kg/day, Institute of Materia Medica, Chinese Academy of Medical Sciences & Peking Union Medical College, Beijing, China) by oral gavage for 28 days.

Study in DIO mice. After 8 weeks induction, DIO mice were randomly divided into two groups, including the model group (DIO) and the CX08005-treated group. Mice were given CX08005 (100 mg/kg/day) by daily oral gavage for 15 days.

During the experimental period, animals were grouped housed (four mice per cage) in grommet cages at room temperature 21~23 °C with a humidity of 40~60%, 12 h light/dark cycle, ad libitum access to water and chow diet. All experiments were approved and performed in accordance with the guidelines for the care and use of laboratory animals, and were approved by the Animal Care Committee of the Peking Union Medical College and the Chinese Academy of Medical Sciences (Beijing, China), (Approval number: 00003338).

### 4.3. Insulin Tolerance Tests

After treatment with CX08005 for 2 weeks, insulin tolerance tests (ITTs) were performed in both KKAy and DIO mice. Mice were fasted for 2 h, then injected subcutaneously with insulin (0.24 U/kg). Blood glucose was measured at 0, 30, 60, and 120 min after insulin injection. The area under the blood glucose-time curve between 0 and 120 min was calculated.

### 4.4. Determination of Triglycerides and Total Cholesterol

The plasma triglycerides (TG) and total cholesterol (TC) were determined in mice. Briefly, blood was collected from the tail vein after fasting 2 h in mice. The contents of TG and CHO were determined by enzymatic colorimetric methods using commercial kits and following the manufacturer’s protocol (BioSino Inc., Beijing, China).

### 4.5. Hepatic Triglycerides Measurement

Hepatic triglycerides were measured following the modified method described by Folch et al. [45]. Briefly, total lipids were extracted from the liver samples by homogenizing the tissues with 4:2:1 chloroform/methanol/PBS (*v*/*v*/*v*) to a final dilution of 70 times the original volume of the tissue sample. The organic layer was then separated, evaporated, and reconstituted in isopropanol. The levels of TG were determined using a colorimetric assay kit (Jian Cheng Bioengineering Institute, Nanjing, China).

### 4.6. Ultrasound Analyses

Liver ultrasound analysis was performed using a method previously validated and used in our laboratory [31]. Briefly, the fasting (2 h) KKAy mice were anesthetized with 0.8% pentobarbital sodium (10 mL/kg body weight). After abdominal hair was shaved, mice were positioned supine on a heated table. A sound conductive gel was applied to the animal and the ultrasound examination was performed with the aid of the VEVO 770 system (VisualSonics, Toronto, ON, Canada) coupled to a 30 MHz probe. Under the condition of 60% contrast, 50% brightness, and 10 db gain, the probe was placed at the 3–4 branches of the left hepatic portal vein. Ultrasound images of two circular regions (0.3 ± 0.02 mm^2^, avoiding blood vessels and bile ducts) of interest (ROI) were taken along the direction of ultrasound propagation of the liver. The average gray value of each area and the distance between the centers of the two areas were then measured.

The hepatic echo-intensity attenuation coefficient was calculated according to the equation:

Hepatic echo-intensity attenuation coefficient = (lnAn − lnAf)/(∆d × f),

where An and Af represent the mean gray value of the near-field and far-field ROIs, respectively; ∆d is the line distance between the two ROIs and f is the ultrasonic probe frequency.

### 4.7. Microcirculation Detection and Parameter Analysis

The hepatic microcirculation in DIO mice was detected with stereomicroscope (DM-IRB, Leica, Germany) as the method previously described [46]. Image data are captured with a color camera (JK-TU53H, 3CCD camera, Toshiba, Tokyo, Japan) and a DVD recorder (DVR-R25, Malata, Zhangzhou, China). The microcirculatory parameters analysis was then performed as the method described previously [47].

### 4.8. Statistical Analysis

Statistical tests were performed by Prisma 5.0 software. All the data are expressed as mean ± SEM. One-way ANOVA analysis followed by the Tukey post hoc test was performed to analyze statistical difference for multiple group comparisons. The *p*-value less than 0.05 was statistically significant.

## 5. Conclusions

Based on the improvement of insulin resistance by inhibiting the target PTP1B, the effects of CX08005 on hepatic steatosis and microcirculation dysfunction, were demonstrated further. As an insulin sensitizer, compound CX08005 can be developed into a well-tolerated potential therapeutic agent for NAFLD.

## Data Availability

The data is contained in the article.
